# Identification of proteins differentially expressed by *Chlamydia trachomatis* treated with chlamydiaphage capsid protein VP1 during intracellular growth

**DOI:** 10.1007/s00203-017-1381-2

**Published:** 2017-04-25

**Authors:** Jingyue Ma, Yina Sun, Changgui Sun, Quan Zhou, Manli Qi, Jie Kong, Jing Wang, Yuanjun Liu, Quanzhong Liu

**Affiliations:** 1 Department of Dermatovenereology, Tianjin Medical University General Hospital, 154 Anshan Road, 300052 Tianjin, China; 2 Key Laboratory of Hormones and Development (Ministry of Health), Metabolic Diseases Hospital and Tianjin Institute of Endocrinology, Tianjin Medical University, 22 Qixiangtai Rd, 300070 Tianjin, China

**Keywords:** *Chlamydia trachomatis*, Chlamydiaphages, VP1, Inhibition

## Abstract

*Chlamydia trachomatis* infection is one of the most prevalent sexually transmitted diseases. Our research pertains to the inhibitory effect and molecular mechanism of the chlamydiaphage capsid protein VP1 on the growth of *Chlamydia trachomatis*. In this research, the capsid protein VP1 of the guinea-pig conjunctivitis chlamydiaphage phiCPG1 was expressed, purified and identified, and then, it was applied to the cultivation of different serovars of *Chlamydia trachomatis* and *Chlamydia psittaci*. The inhibitory effect was observed in each serovar of *Chlamydia trachomatis* (D, E, F, G, H, I, K, and L2) and *Chlamydia psittaci* inoculated with VP1 protein. The inhibition affection of VP1 on the growth of *Chlamydia trachomatis* was caused by the changes of expressions of some related proteins including 36 proteins up-regulated and 81 proteins down-regulated in the development cycle of Ct through the label-free test, and the transcription levels of these proteins, including Hc1, pmpD, and MOMP, were confirmed by RT-PCR. It provides information that is essential for understanding the mechanism of chlamydiaphage capsid protein VP1 on chlamydia and a new direction for further clinical treatment of chlamydial infection.

## Introduction

*Chlamydia trachomatis* (Ct) is one of the pathogens that commonly occur in human reproductive tract infections, accounting for 40–50% of the world’s most prevalent sexually transmitted diseases (Marrazzo and Suchland 2014). Chronic chlamydial disease may lead to serious sequelae, including pelvic inflammatory disease, ectopic pregnancy, infertility, and trachoma—the world’s leading cause of preventable blindness (Kohlhoff and Hammerschlag 2015). Resistance to chemotherapeutic agents is widespread, and consequently, the focus of the study of *Chlamydia trachomatis* has been enlarged to the chlamydiaphage in recent years (Śliwa-Dominiak et al. 2013).

Six different phages of chlamydiae have been described and characterized during the past several years (Śliwa-Dominiak et al. 2013; Sait et al. 2011). The chlamydiaphages remain with a class of lytic phages for which the single-stranded DNA coliphage X174 is the typical example. The chlamydial host range for different chlamydiaphages is varied, but all are lytic for their respective hosts. Nevertheless, it is interesting to note that chlamydiaphages have not yet been discovered in association with *Chlamydia trachomatis*. Hsia et al. once studied the activity of phiCPG1 against *Chlamydia caviae* strain guinea-pig inclusion conjunctivitis (GPIC) and revealed a significant inhibitory effect of phiCPG1 on GPIC by inducing abnormally large reticulate bodies (RBs) (Hsia et al. 2000a, b).

In a recent research of the corresponding analysis of chlamydiaphages, the well-conserved VP1 which is the main structure protein on viral envelope has a surface-exposed 71- to 85-amino-acid residue loop and was predicted to contain a latent receptor binding site (Garner et al. 2004; Read et al. 2000; Everson et al. 2003). It may play an important role on phage adhesion and penetration of Chlamydiae.

The purpose of the subject study was to find whether VP1 of chlamydiaphage phiCPG1 has an effect on the growth of *Chlamydia trachomatis* and GPIC and ascertain the corresponding mechanism. Specifically, recombinant VP1 from phiCPG1 in GPIC was generated and purified. Then, this protein was incubated with GPIC and *Chlamydia trachomatis* throughout the culturing period. The resulting effect of VP1 on the growth of *Chlamydia trachomatis* and GPIC was observed. Subsequently, the RBs of *Chlamydia trachomatis,* after being treated with or without VP1 protein, were detected by means of the label-free test, and some of the differentially expressed proteins were confirmed by RT-PCR.

## Materials and methods

### Expression, purification, and quantification of recombinant VP1

The VP1 protein was expressed, purified, and quantified as described previously (Guo et al. 2016). The quantified VP1 protein was aliquoted and stored in freezer for the following experiment.

### The effect of VP1 on *Chlamydia trachomatis* and GPIC

The frozen standard strains of *Chlamydia trachomatis* or GPIC were thawed, after which they were augmented with equivoluminal PBS, 50 ug/mL BSA in PBS, or purified VP1 protein in PBS, thus achieving a final concentration of 50 ug/mL (Liu et al. 2012).The strains were incubated at room temperature for 3 h to ensure that the VP1 protein would have adequate contact with *Chlamydia trachomatis* or GPIC. The same mixture, in the same three respective amounts, was added to the single density McCoy cells in 24-well plates. These McCoy cell plates were centrifuged for 1 h, and after 2 h, the fluid was exchanged for new fluid with the same respective volumes of PBS, BSA, and VP1 protein, the final concentration of which was also 50 ug/mL. After 48 h in an incubator at 37 °C, the liquid was discarded and the cells were allowed to dry naturally, after which they were fixed with methanol for 10 min at room temperature and iodine-stained. We counted the numbers of inclusions of every Ct, including D, E, F, G, H, I, K, and L2, obtaining an average of 30 fields of microscope’s view (Guo et al. 2016). We also observed the inclusions of GPIC by fluorescent microscopy using polyclonal antibodies (anti-EB serum 1:1000) and secondary antibody of goat anti-rabbit-FITC (1:80) (Solarbio, China).

### The purification of EB and RB of *Chlamydia trachomatis*

VP1 protein was added to the six-well plate containing dense, single-layer McCoy cell containing pure E standard strains of *Chlamydia trachomatis* with 50, 40, 30, 20, 10, and 0 ug/mL VP1 concentration. As doing in the above, 48-h post-infection (hpi) later the cultivated results were observed under an inverted microscope. We collected two kinds of McCoy cells inoculated with serovar E standard strain of *Chlamydia trachomatis* in equivoluminal PBS cultivated with or without 10 ug/mL VP1 protein. These cells were centrifuged at 1200 rpm for 20 min. In the biosafety cabinet, each sample was sonicated at 19%, three times for 2.5 min at an interval of 4 s. The broken cells and activity of chlamydial elementary bodies (EBs) were observed by microscope. The supernatant was transferred to a fresh centrifuge bottle of 35% Hypaque-76 (Lunan pharmaceutical, China) and centrifuge at 16,000 rpm for 60 min at 4 °C. The supernatant was gently decanted, after which the pellets were resuspended in 5 mL ice-cold SPG and placed onto the upper fluid of Hypaque-76 with gradients of 40, 44, and 52%. The centrifuge tubes were loaded into swinging buckets, and the tubes were balanced. The sample was centrifuged at 17,000 rpm for 90 min at 4 °C. The EBs were removed from the 52/44% Hypaque-76 interface with a clean cannula, and the RBs should be removed from 44/40% Hypaque-76 interface. The tubes were washed with SPG, and the EBs or RBs were collected after centrifuging at 14,000 rpm for 30 min at 4 °C, after which they were kept at −80 °C.

### Differentially expressed protein of Ct treated with VP1 protein by label-free test

#### Protein Extraction

The purified *Chlamydia trachomatis* RBs pellet was lysed in 2% SDS at 97 °C and then sonicated with 12 short bursts of 10 s each, followed by intervals of 30 s for cooling. Unbroken cells and debris were removed by centrifugation at 4 °C for 10 min at 20 kg. The protein content in the supernatant was determined with the 2D Quant Kit (GE Healthcare) according to the manufacturer’s instructions. Protein of equal amount was loaded and separated in 12% SDS-PAGE gel. When bromophenol blue band entered the separation gel in excess of 1 cm, the electrophoresis was stopped and the gel was stained with Coomassie Brilliant Blue G-250.

#### In-gel digestion and peptide extraction

After destaining, the gel piece above the bromophenol blue position was cut into small cubes of 1 mm^3^, which were washed three times with 500 uL H_2_O and destained with 500 uL of 100 mM ammonium bicarbonate in 50% acetonitrile on a 50 °C thermo mixer. The gel pieces were dehydrated with 500 uL acetonitrile. Disulfide bond was cleaved by incubating gel pieces for 60 min at 56 °C with 200 uL of 10 mM DTT in 100 mM ammonium bicarbonate. Alkylation of the cysteines was performed by incubating for 45 min at room temperature in darkness with 200 uL of 55 mM iodoacetamide in 100 mM ammonium bicarbonate. The gel pieces were covered with trypsin solution (10 ng/uL in 100 mM ammonium bicarbonate). After 10 min of incubation on ice, the remaining trypsin solution was removed and the appropriate amount of 100 mM ammonium bicarbonate was added to cover the gel pieces. Proteolysis was performed overnight at 37 °C and stopped by adjusting the samples to 2% formic acid. The peptide in gel was extracted once by 200 uL 0.1% formic acid in 50% acetonitrile and twice by 200 uL 0.1% formic acid in 100% acetonitrile.

#### LC–ESI–MS/MS Analysis by Q Exactive Plus

The peptide was vacuum-dried in ScanVac MaxiVac Beta (Labogene), resuspended in buffer A (2% ACN, 0.1% FA), and centrifuged at 2 kg for 2 min. The supernatant was transferred into a sample tube and loaded onto an Acclaim PepMap 100 C18 trap column (Dionex, 75 um × 2 cm) by UltiMate 3000 Nano LC (Dionex), whereupon the peptide was eluted onto an Acclaim PepMap RSLC C18 analytical column (Dionex, 75 um × 25 cm). A 70-min gradient was run at 300 nl/min, starting from 8% and proceeding to 35% B (80% ACN, 0.1% FA), followed by 3 min of linear gradient to 80% B, and maintenance at 80% B for 5 min. The peptides were subjected to NSI source followed by tandem mass spectrometry (MS/MS) in Q Exactive plus (Thermo Scientific) coupled online to the UPLC. Intact peptides were detected in the orbitrap at a resolution of 70,000. Peptides were selected for MS/MS using 27% NCE, and ion fragments were detected in the orbitrap at a resolution of 17,500. A data-dependent procedure that alternated between one MS scan followed by 15 MS/MS scans was applied for the top 15 precursor ions above a threshold ion count of 1E5 in the MS survey scan with 30 s of dynamic exclusion. The electrospray voltage applied was 1.8 kV. Automatic gain control (AGC) was used to prevent the overfilling of the ion trap; 3E6 and 1E5 ions were accumulated for the generation of MS and MS/MS spectra separately. For MS scans, the *m*/*z* scan range was 350–1500 Da.

#### Database search

Protein identification was performed by means of the MaxQuant software with integrated Andromeda search engine (v1.3.0.5). The tandem mass spectra were searched against *Chlamydia trachomatis* serovar E (strain E/11,023) UniProt reference proteome database (926 sequences) concatenated with a reverse decoy database and protein sequences of common contaminants. Trypsin/P was specified as the cleavage enzyme allowing up to two missing cleavages, three modifications per peptide, and five charges. Mass error was set to 6 ppm for precursor ions and 0.02 Da for fragment ions. Oxidation on Met and acetylation on protein N-terminal were specified as variable modifications. The FDR (False Discovery Rate) thresholds for protein, peptide, and modification site were specified as 0.01. The minimum peptide length was set as 6.

#### Protein quantification

The label-free quantification for identified proteins was performed with the Max LFQ software integrated into the MaxQuant software. In addition to the parameter settings mentioned above, LFQ quantification was enabled by checking “Match between runs,” “LFQ”, and “iBAQ.” All the other parameters in MaxQuant were set to their default values. After MaxQuant processing, the proteinGroup.txt file was loaded and analyzed by the Perseus software (v1.5.0.31). Briefly, the LFQ intensity values of two samples were transformed logarithmically. The missing LFQ value was replaced by normal distribution to simulate the background LFQ intensity level for undetected protein abundance values. The relative protein ratio was calculated by exponentially transformation of difference value between logarithmically transformed LFQ values of two samples.

### RT-PCR detection of differentially expressed genes

We selected three genes for quantitative reverse transcription–polymerase chain reaction (RT-PCR) for validation of the data detected by label-free test. The primers of these three genes and housekeeper gene-tufA were listed in the Table 1. Total RNA was isolated from McCoy cells infected *Chlamydia trachomatis* serovar E at 0, 12, 24, 36, and 48 hpi in the presence or absence of 10 ug/mL VP1 using the Trizol reagent (Tiangen Biotech, China) according to the manufacturer’s instructions.

For reverse transcription, 3 μg of total RNA was used for creation of single-stranded cDNA using reverse transcriptase M-MLV (Promega, Madison, WI, USA) in 20 μl. PCR was carried out with 1 μl of the RT products as follows: initial denaturation at 94 °C for 2 min followed by 40 cycles of 94 °C for 15 s, 55 °C for 15 s, and 72 °C for 25 s, followed by a final elongation at 72 °C for 8 min. The transcription level of RT-PCR results of Ct growth-related genes was analyzed by means of the quantitative comparison of Ct (threshold cycles), which was calculated by 2^−ΔΔCt^ at the same timepoint, because the amplification efficiency E of the purpose gene and housekeeping gene was nearly 100%. Based on tufA for housekeeping gene, Δ Δ Ct = (Ct purpose gene − Ct tufA) VP1 Ct group − (Ct purpose gene − Ct tufA) Ct group and the quantity of purpose gene = 2^−ΔΔCt^. If the quantity of relative purpose gene >1, transcription is increased for that purpose gene, whereas if the quantity of the relative purpose gene <1, transcription is decreased for that purpose gene. At least three PCR assays were performed for each timepoint using each gene-specific primer set each time.

### Data analysis

All of the data were analyzed with the SPSS 17.0 software (IBM, Armonk, NY, USA) according to the statistics. The difference was considered statistically significant if the *p* value was less than 0.05.

## Results and discussion

### The effect of VP1 on *Chlamydia trachomatis*

**Fig. 1:**
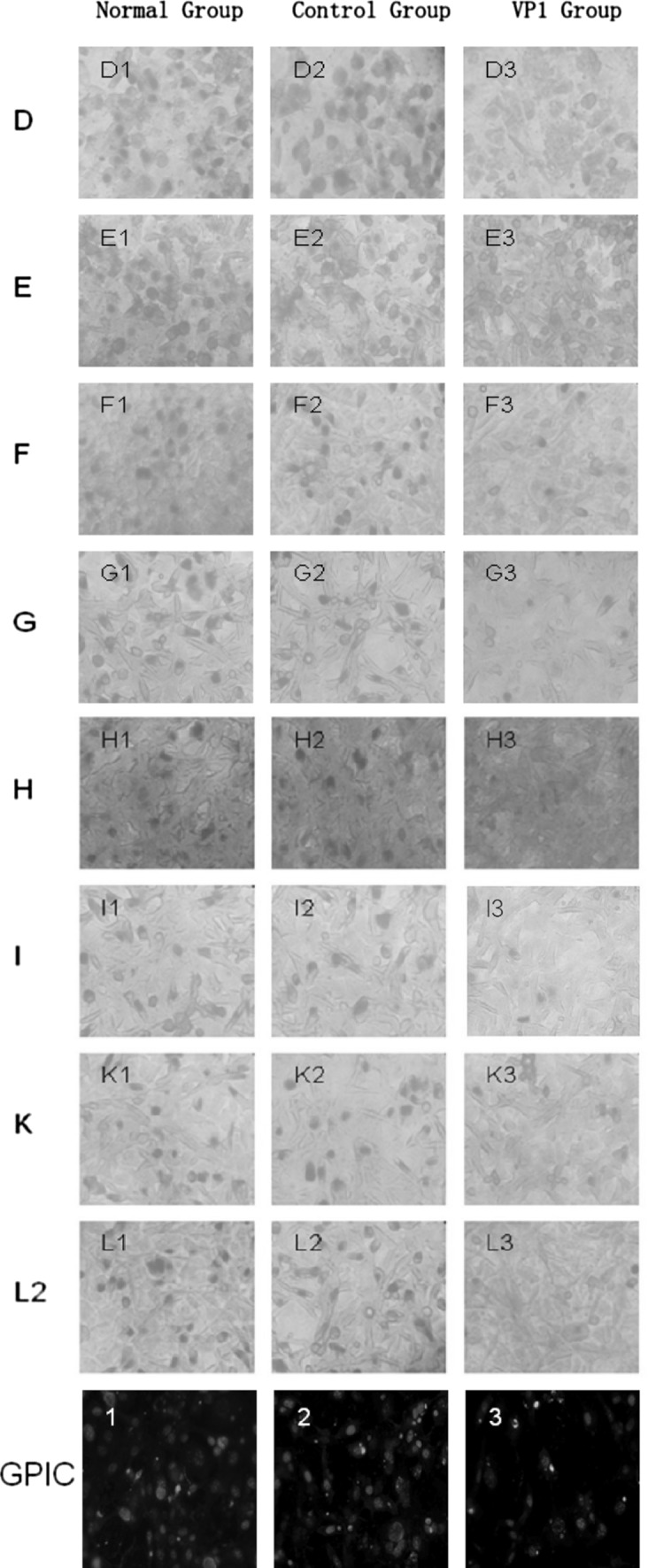

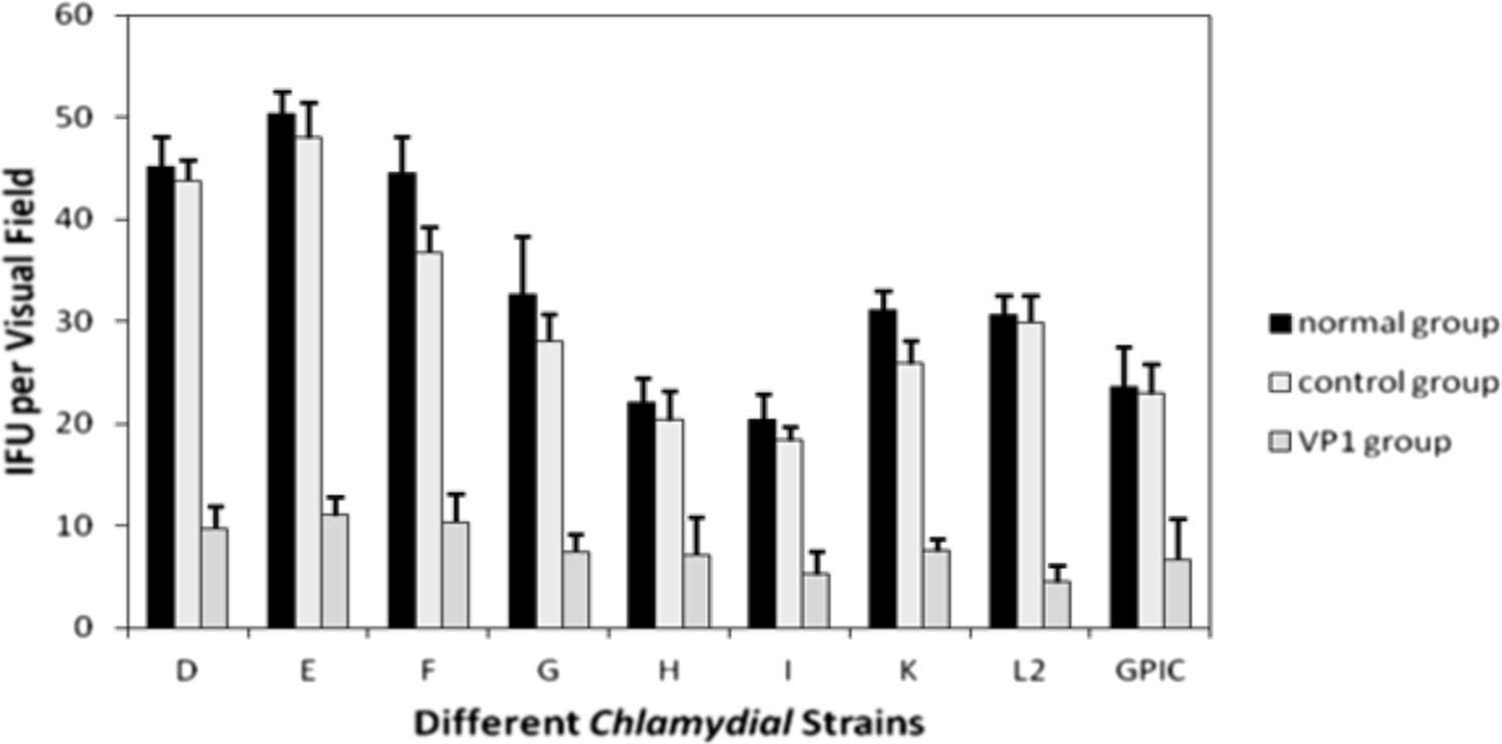
Inhibition effect of VP1 on the growth of different Ct strains and GPIC. (*1*) Different Ct strains (**D**, **E**, **F**, **G**, **H**, **I**, **K**, **L2**) or GPIC strain was preincubated with PBS (*normal group*), BSA in PBS (*control group*), or purified VP1 in PBS (*VP1 group*), and then, these mixtures were inoculated into the HeLa cells with PBS (*D1*, *E1*, *F1*, *G1*, *H1*, *I1*, *K1*, *L1*, *1*), BSA in PBS (*D2*, *E2*, *F2*, *G2*, *H2*, *I2*, *K2*, *L2*, *2*), or purified VP1 in PBS (*D3*, *E3*, *F3*, *G3*, *H3*, *I3*, *K3*, *L3*, *3*). After 48 h, HeLa cells with the different Ct strains were stained with iodine and those with GPIC were used for double immunofluorescence labeling (1–3) for chlamydial organisms (*green*) and DNA (*blue*). Note that VP1 has an obviously inhibitive effect on the growth of *Chlamydia trachomatis* and GPIC. (*2*) Bar graph shows the obvious inhibition effects of VP1 on different Ct strains from **D** to **L2** and **GPIC** (color figure online)

It has been reported that chlamydiaphages inhibit the Chlamydia replication and physiological process so that the chlamydia RBs cannot develop into mature EBs (Pawlikowska-Warych et al. 2015). The process by which PhiCPG1 infects *Chlamydia caviae* can be observed through transmission electron microscope: the size of chlamydial inclusion bodies with phage infection was smaller than that without chlamydiaphage 24–32 h post-infection, and RBs abnormally increased two to five times also appeared in the chlamydial inclusion body with phage infection (Hsia et al. 2000a, b). In our study *Chlamydia trachomatis* was pretreated and sufficiently combined with VP1 protein before inoculation, which was similar to the infection process of chlamydiaphages, and was then treated with the VP1 protein throughout the development cycle (Liu et al. 2012). VP1 protein may play a similar but inferior role with chlamydiaphages by inhibiting the development of RBs, so that they would not become mature EBs (Guo et al. 2016). The VP1 protein has obvious inhibition to the Ct at 3 × 10^5^ IFU/ml treated with VP1 of the concentration 50 ug/ml. Compared with the normal growth of Ct, the inhibition rates (IR) of VP1 on the standard strains of Ct D, E, F, G, H, I, K, and L2 were, respectively, 78.30 ± 1.91, 77.90 ± 0.76, 76.40 ± 1.45, 76.70 ± 1.92, 68.90 ± 2.10, 73.50 ± 2.85, 75.60 ± 1.72, and 85 ± 1.57% (Fig. 1). Although IR showed a difference among Ct strains, the difference was not significant (*P* > 0.05). As VP1 is the capsid protein of the chlamydiaphage phiCPG1, which is the phage of GPIC, a strain of *Chlamydia psittaci*, we assessed the effect of VP1 protein on GPIC. Compared with the Ct groups, although IR was higher in GPIC group (80.2%), the difference has no statistical significance (*P* > 0.05). Our results demonstrated that the VP1 protein could significantly inhibit the growth of *Chlamydia trachomatis*.

**Table 1:** Primer sequences for real-time polymerase chain reaction

Name	Accession number	Sense primer sequences	Antisense primer sequences	Product length (bp)
tufA	BOUR_00335	5′-AACGTGGGAAGCGTTAATTG-3′	5′-GGTTGGCTGATTTTCGTGAT-3′	87
MOMP (ompA)	BOUR_00729	5′-CCTGCTGAACCAAGCCTTAT-3′	5′-TGATAGCGTCACACCAAGTG-3′	95
Hc1	BOUR_00796	5′-TACGAACTCTTTGTATG-3′	5′-TGACTGACTTGTTGGAAA-3′	82
pmpD	BOUR_00871	5′-CTTGATTCTCCTCGTGAC-3′	5′-TGTGATTCCAGCCTTACT-3′	76

**Fig. 2:**
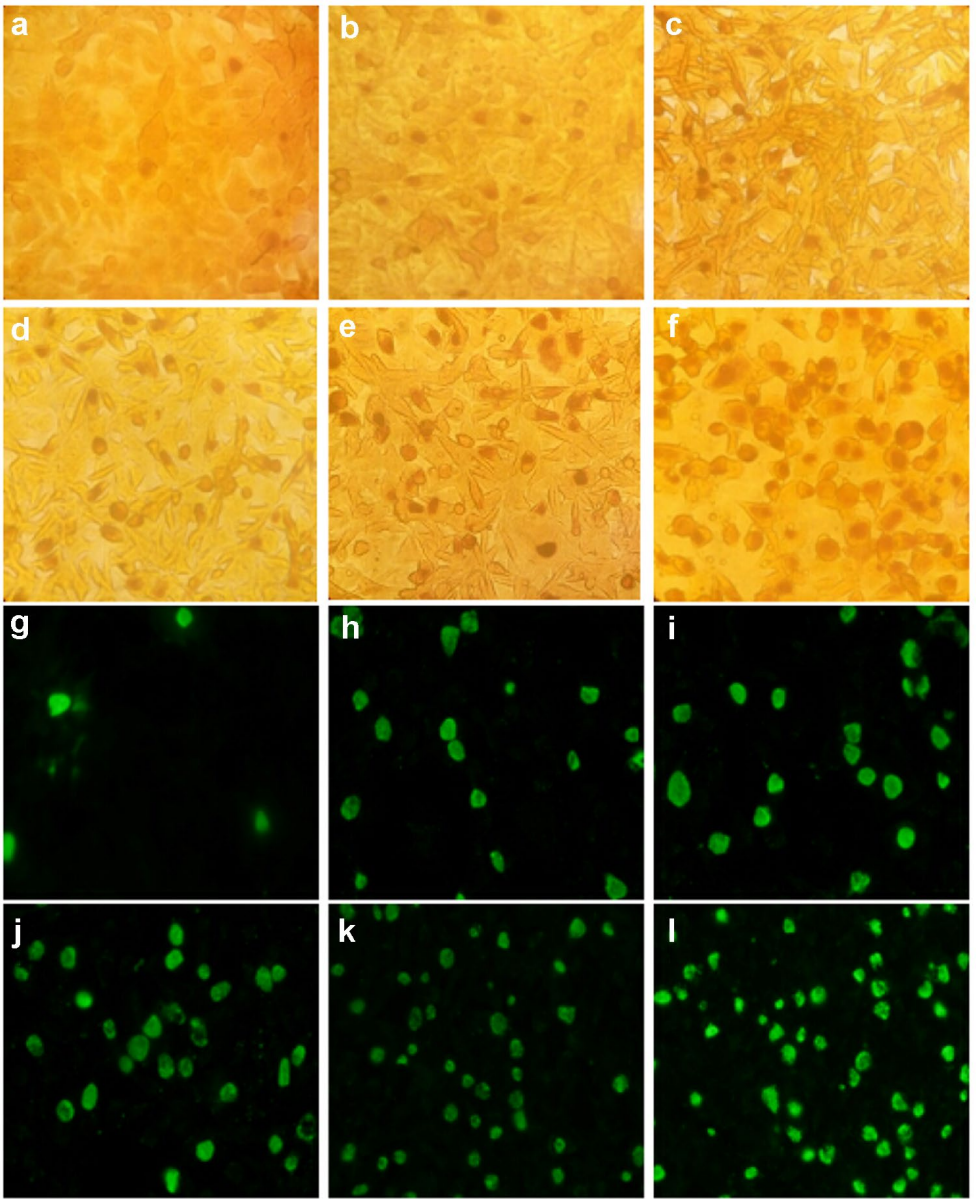
Inhibition effect of VP1 with different concentrations on *Chlamydia trachomatis* serovar E. The Ct serovar E was treated with VP1 from 50 ug/mL (**a**, **g**), 40 ug/mL (**b**, **h**), 30 ug/mL (**c**, **i**), 20 ug/mL (**d**, **j**), 10 ug/mL (**e**, **k**) to 0 ug/mL (**f**, **l**) following the method described in the Fig. 1; after culturing for 48 h, the iodine staining (**a**–**f**) and immunofluorescent staining (**g**–**l**) were used to observe the result. With the reduction of VP1 concentration, the number of inclusions increased significantly

Because a high proportion of clinical strains of Ct infection is serovar E, we chose standard-strain E as the following experiments strain. With reduction of VP1 concentration to E standard strains (50, 40, 30, 20, 10, and 0 ug/mL), the number of inclusions increased significantly (11.05 ± 2.21, 13.48 ± 2.15, 20.56 ± 1.75, 27.34 ± 1.97, 32.64 ± 1.19, and 49.17 ± 2.35). The difference between the results of the two groups of 10 and 0 ug/mL (*P* < 0.05) was statistically significant (Fig. 2). We then chose the VP1 concentration of 10 ug/mL in subsequent experiments.

### Differentially expressed proteins in *Chlamydia trachomatis* treated with VP1 by label-free test

We chose the concentration of 10 ug/mL of VP1 for the subsequent experiments, because, at such a concentration, we could obtain sufficient RBs after the VP1 effect, and the differences would be statistically significant. RBs purified from serovar E of standard-strain Ct with or without VP1 protein were detected by means of the label-free protein quantitative test. Consequently, the number of quantitative differentially expressed proteins was 583, among them the number of proteins down-regulated with respect to *Chlamydia trachomatis* serovar E treated with 10 ug/mL VP1 was 81, but the number of proteins up-regulated was 36 (Tables 2, 3). Among them, the down-regulated proteins of histone H1-like developmental protein (the protein ID: ADH21233), the major outer membrane protein (the protein ID: ADH21166), and the up-regulated protein of the polymorphic outer membrane protein (the protein ID: ADH21310) were chose to do the conformation experiment.

**Table 2:** Protein down-regulated of *Chlamydia trachomatis* serovar E treated with VP1

Protein IDs	The name of proteins	Ratio
ADH21371	Glycogen branching enzyme	0.49704928
ADH21299	30S ribosomal protein S18	0.492113887
ADH21160	ATP: guanido phosphotransferase	0.491899232
ADH21122	Exodeoxyribonuclease V beta chain	0.488969166
ADH21091	Dihydrofolate reductase	0.486130459
ADH20978	Nucleoside diphosphate kinase	0.481022312
ADH21309	Putative glycerol-3-phosphate acyltransferase PlsX	0.480790883
ADH21077	Translocation protein TolB	0.479146122
ADH20730	*N*-Acetylmuramoyl-l-alanine amidase	0.47183401
ADH21327	CDP-diacylglycerol–serine O-phosphatidyltransferase	0.471713009
ADH21232	rRNA methyltransferase	0.470958386
ADH21064	Tryptophanyl-tRNA synthetase	0.468193909
ADH20874	dnaK suppressor protein	0.465642613
ADH21161	Hypothetical protein	0.459496009
ADH21089	Hypothetical protein	0.457619072
ADH20841	Hypothetical protein	0.452647713
ADH20630	Tryptophan synthase subunit alpha	0.449188885
ADH20867	Branched-chain alpha-keto acid dehydrogenase subunit E2	0.43448508
ADH21314	Hypothetical protein	0.434428216
ADH20990	30S ribosomal protein S5	0.427995964
ADH20812	Ribonuclease Z	0.419154485
ADH21014	DNA polymerase III subunit epsilon	0.418368326
ADH20932	Ribosomal-protein-alanine acetyltransferase	0.416961914
ADH20723	DNA polymerase III subunit epsilon	0.402722135
ADH20484	50S ribosomal protein L19	0.396907281
ADH20719	Putative cysteine desulfurase	0.395167393
ADH20986	30S ribosomal protein S11	0.394762187
ADH20911	Carboxy-terminal processing protease	0.368370849
ADH21072	Putative hydrolase	0.367335456
ADH20759	Hypothetical protein	0.364758481
ADH21258	tRNA delta(2)-isopentenyl pyrophosphate transferase	0.36412152
ADH20541	Hypothetical protein	0.363236349
ADH20695	Crp-family transcriptional regulator	0.361308099
ADH21315	Phosphoglucosamine mutase	0.361051678
ADH20590	ABC transporter, ATP-binding component	0.359046248
ADH21297	Peptidyl-tRNA hydrolase	0.357898872
ADH20752	Hypothetical protein	0.35784115
ADH21166	Major outer membrane protein	0.35012415
ADH21112	CpxR	0.344958042
ADH21172	Hypothetical protein	0.34421956
ADH20468	Hypothetical protein	0.339660504
ADH20566	Hypothetical protein	0.328301035
ADH20464	Ribonuclease HIII	0.322986324
ADH21223	Putative lipoprotein	0.313085136
ADH21313	Hypothetical protein	0.303897406
ADH20961	Hypothetical protein	0.300454165
ADH20785	Translation initiation factor IF-1	0.288869616
ADH20748	ATP-dependent Clp protease	0.284460131
ADH21046	Hypothetical protein	0.278999925
ADH21331	Putative SAM-dependent methyltransferase	0.27664151
ADH21257	Anti-sigma F factor antagonist	0.259617334
ADH20735	Hypothetical protein	0.245592846
ADH20977	Lipoate-protein ligase A	0.244497189
ADH21100	Hypothetical protein	0.242251971
ADH21233	Histone H1-like developmental protein	0.24223916
ADH21004	50S ribosomal protein L23	0.239435955
ADH21013	Acyl-CoA hydrolase	0.209805678
ADH20889	Hypothetical protein	0.209802349
ADH21176	ABC transport protein, ATPase component	0.208192025
ADH20749	tRNA-specific 2-thiouridylase MnmA	0.205210925
ADH20729	Integration host factor alpha subunit	0.204808434
ADH20607	Hypothetical protein	0.201780525
ADH20580	Ribulose-phosphate 3-epimerase	0.184262145
ADH20545	4-Alpha-glucanotransferase	0.181454012
ADH21285	Excinuclease ABC subunit C	0.179283568
ADH21051	General-secretion-pathway protein D	0.178362725
ADH20647	Thymidylate kinase	0.168508907
ADH20744	Glycine cleavage system protein H	0.141533402
ADH20669	Glutamate-1-semialdehyde aminotransferase	0.127305884
ADH21078	Peptidoglycan-associated lipoprotein	0.126172525
ADH20611	Lipoprotein releasing system, inner membrane component	0.120328257
ADH20847	Metal-dependent hydrolase	0.111782193
ADH20727	Acetyl-CoA carboxylase carboxyltransferase subunit alpha	0.093594725
ADH21195	SWF/SNF-family helicase	0.088404008
ADH20543	Putative decarboxylase	0.085352845
ADH21360	Sulfate transporter	0.082860148
ADH21036	Lipoyl synthase	0.079090553
ADH20817	Hypothetical protein	0.077451694
ADH21056	Hypothetical protein	0.066584912
ADH20793	Exodeoxyribonuclease VII large subunit	0.033123691
ADH21031	Putative methyltransferase	0.003636102

**Table 3:** Potein up-regulation of *Chlamydia trachomatis* serovar E treated with VP1

Protein IDs	The name of proteins	Ratio
ADH20816	Putative lipoprotein	15.07549086
ADH20765	Hypothetical protein	11.35416606
ADH21226	SET domain containing protein	9.577652983
ADH21286	DNA mismatch-repair protein MutS	9.009897518
ADH21128	Integral membrane protein	8.672221902
ADH20564	Pseudouridine synthetase-family protein	8.220523933
ADH21025	Hypothetical protein	8.169375254
ADH20473	Hypothetical protein	7.607968196
ADH20558	4′-Phosphopantetheinyl transferase	7.225913234
ADH20942	Two component system response regulator	6.37518932
ADH20688	Hypothetical protein	5.40791301
ADH21092	2-Amino-4-hydroxy-6-hydroxymethyldihydropteridine pyrophosphokinase	4.895461188
ADH21050	General-secretion-pathway protein E	4.832609268
ADH20903	Uroporphyrinogen-III synthase	4.57054323
ADH20813	Site-specific tyrosine recombinase XerC	4.451198621
ADH21271	Primosome assembly protein PriA	4.438716816
ADH21351	Hypothetical protein	4.135269236
ADH21247	60 kDa chaperonin GroEL	4.019083233
ADH21253	Undecaprenyldiphospho-muramoylpentapeptide beta-*N*-acetylglucosaminyltransferase	3.584225615
ADH21087	DNA helicase	3.483931842
ADH20472	Hypothetical protein	3.359168339
ADH21332	UDP-*N*-acetylenolpyruvoylglucosamine reductase	3.231605756
ADH21123	Exodeoxyribonuclease V gamma chain	3.101551076
ADH21228	Cell-division protein	2.970758436
ADH20506	Hypothetical protein	2.95220001
ADH20534	DNA polymerase III beta subunit	2.858081534
ADH21152	Hypothetical protein	2.679738898
ADH20605	Serine/threonine-protein kinase PKN1	2.628586315
ADH20838	Bifunctional 3-dehydroquinate dehydratase/shikimate dehydrogenase protein	2.480453816
ADH20885	GTPase ObgE	2.414902641
ADH21140	2-Dehydro-3-deoxyphosphooctonate aldolase	2.366285718
ADH20756	Acetyl-CoA carboxylase beta subunit	2.262933275
ADH21236	Coproporphyrinogen III oxidase	2.244083334
ADH21310	Polymorphic outer membrane protein	2.217780394
ADH21084	Putative deoxyribonucleotide triphosphate pyrophosphatase	2.210906572
ADH21188	Hypothetical protein	2.04696355

### Transcriptional analysis of selected genes

According to the reference sequence of investigated gene in Ct growth, we designed the upstream and downstream primers of Hc1, MOMP, pmpD, and housekeeper gene-tufA. TufA can encode the extension factor EF-Tu, including protein synthesis, as it is not only well expressed throughout development but is also a reliable measure of exponential growth, particularly of Ct index growth, which can be used as our RT-PCR reference-housekeeper gene (Carrasco et al. 2011). Because EF-Tu is one of the most important factors in protein biosynthesis, data generated from further studies of EF-Tu should help to clarify events in the developmental cycle of *Chlamydia* spp. (Zhang et al. 1994).

**Fig. 3:**
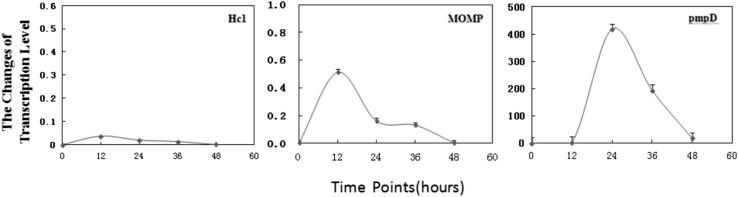
Real-time polymerase chain reaction analysis of transcription level of investigated genes (Hc1, MOMP, and pmpD). Transcription levels were measured by real-time PCR during the developmental cycle of *C. trachomatis* treated with or without 10 ug/mL VP1 at 0, 12, 24, 36, and 48 h after infection. Transcription level of the tufA gene encoding EF-Tu was used for comparison. The 2^−ΔΔCt^ of the transcription level of pmpD > 1, indicating that the transcription of pmpD was increased at different timepoints after VP1 treatment, whereas 2^−ΔΔCt^ of the transcription level of MOMP or Hc1 < 1, indicating that transcription of these two genes was decreased. The changes of the transcription level of pmpD, MOMP, or Hc1 were consistent with the changes of the protein level detected by label-free quantitative test

The transcription levels of chlamydial genes in the presence or absence of 10 ug/ml VP1 could be analyzed by means of RT-PCR. Temporal expression of Hc1, MOMP, and pmpD in the presence or absence of 10 ug/ml VP1 was determined by infecting McCoy cells at 3 × 10^5^ IFU/ml and collecting samples for RNA isolation at 0, 12, 24, 36, and 48 hpi (Fig. 3). The reduced-transcription genes treated with VP1 are MOMP and Hc1, while the increased-transcription gene treated with VP1 is pmpD. There were fluctuations of the transcript levels during the development cycle about pmpD and MOMP as demonstrated in Fig. 3. The gene-transcription levels of pmpD, MOMP, and Hc1 were consistent with the protein expression results detected by label-free quantitative test.

The stability of chromatin of chlamydial EBs is maintained by the chlamydial histone H1 homologues (Hc1 and Hc2), and therefore, these two proteins globally control chlamydial gene expression. These two histone H1 homologues not only play an important part in foundation of a nucleoid construction but also in the down-regulation of genetic expression. The influence of Hc1 on genetic expression styles demands that chlamydiae entirely manage Hc1 activity (Grieshaber et al. 2006). Thus, the activity of Hc1 gene is managed in time of transcription. On account of the powerful regulatory and configurational effects of Hc1 exertion, its expression seems to be regulated at numerous standards, as has been shown previously. Through 6 hpi, Hc1 protein levels kept relatively quantity, but reduced dramatically by 12 hpi, probably because of degradation. New Hc1 protein translation came out when RBs start to distinguish into EBs between 12 and 24 hpi (Tattersall et al. 2012). According to our research results, we supposed that VP1 protein might result in the down-regulation of the expression of Hc1 to inhibit RBs develop into EBs.

pmpD is located in the surface of chlamydial reticulate body and is then secreted after cracking. It is conjectured that pmpD may plays a role in the transformation of RBs into infectious EBs. The deficiency of pmpD intruded upon the membrane alliance of RBs’ inclusion (Taylor et al. 2011). Therefore, pmpD, which takes part in the inchoate interactions between the chlamydia and host cells, is a crucial virulence factor. It was reported that anti-pmpD antibody could issue in the neutralization of EBs and decrease its infectiousness in vitro, and pmpD is thus thought to interact with the host antigen so as to inhibit Ct growth and development (Kari et al. 2014). It was also reported that recombinant pmpD—M13 phage can significantly reduce the number and size of chlamydial inclusion body, and thus, it appears that PmpD plays a direct role in ameliorating Ct infection (Bhattarai et al. 2012). In our study, VP1 could increase the expression of pmpD in Ct and we supposed that the effect of VP1 on the growth of Ct was mediated by pmpD, but the precise mechanisms involved will require further study.

The OmpA family located in the cell outer membrane is a series of proteins having genetically relevant, heat-qualifiable, surface-exposed porin. These proteins play significant pathogenic effects: bacterial conglutination, invasion, intracellular surviving existence, and the ability to evade host defenses and to stimulate pro-inflammatory cytokine production. As well as MOMP can be worked as the immune system targets with immunogenicity concerned in molecule surface-exposed loops. Under many circumstances, MOMP proteins are being assessed as possible vaccine candidates (Confer and Ayalew 2013). In *Chlamydia trachomatis,* major outer membrane protein (MOMP), accounting for approximately 60% of the outer membrane whose function as a structural protein that contributes to the rigidity of the chlamydial elementary body, has weak anion selectivity and ATP permeability and is the main channel to take ATP in the RB host (Danilition et al. 1990; Wyllie et al.^1998^). The means by which VP1 decreases the Ct infection rate may block ATP from the intake channel by decreasing MOMP expression.

In summary, Guinea-pig conjunctivitis chlamydiaphage phiCPG1 capsid protein VP1 has obvious inhibitory effect to *Chlamydia trachomatis* growth. We found the treatment of VP1 to serovar E standard strain of *Chlamydia trachomatis*, caused the expression changes of some related proteins in chlamydia and these differentially expressed proteins may affect the chlamydial growth. It provides important information for understanding the mechanism behind the action of chlamydiaphage capsid protein VP1 against *Chlamydia trachomatis*, which provides a new direction for further study concerning the application of chlamydiaphages in the clinical treatment of *Chlamydia trachomatis*.
